# Integrating nutrition outcomes into agriculture development for impact at scale: Highlights from the Canadian International Food Security Research Fund

**DOI:** 10.1111/mcn.12812

**Published:** 2019-05-31

**Authors:** Annie S. Wesley, Renaud De Plaen, Kristina D. Michaux, Kyly C. Whitfield, Timothy J. Green

**Affiliations:** ^1^ Agriculture and Food Security Program International Development Research Centre Ottawa Ontario Canada; ^2^ Food, Nutrition and Health University of British Columbia Vancouver British Columbia Canada; ^3^ Department of Applied Human Nutrition Mount Saint Vincent University Halifax Nova Scotia Canada; ^4^ Department of Pediatrics and Reproductive Health University of Adelaide Adelaide South Australia Australia; ^5^ Healthy Mothers, Babies, Children Research Theme South Australia Health and Medical Research Institute, Women's and Children's Hospital Adelaide South Australia Australia

**Keywords:** children, food and nutrition security, nutrition education, nutrition‐sensitive agriculture, scaling up, women

## Abstract

The Canadian International Food Security Research Fund programme supported research and scaling up of nutrition‐ and gender‐sensitive agriculture innovations from 2009 to 2018. Women and girls were identified as agents of change and were targeted as the main programme beneficiaries. Projects were implemented in 25 countries through multistakeholder partnerships among universities, research institutions, public and private sectors, and civil society groups, reaching over 78 million people, mainly women and children. Approaches specific to nutrition included growing more nutritious crops, improving dietary diversity, value added processing, food fortification, and nutrition education. Scale‐up for impact was achieved through a number of pathways that started with evidence through rigorous research, followed by a combination of elements such as understanding local and regional contexts to identify specific bottlenecks and opportunities for the deployment and adoption of successful innovations, selecting politically effective or influential partners to lead the scaling up process, and investing in long‐term local capacity and leadership building. Overall, the knowledge generated in the programme indicate that well‐designed nutrition‐sensitive agriculture and food‐based interventions can have meaningful impacts on pathways that will lead to better health and well‐being of women and children through improving household and individual access to nutrient‐rich foods. Longer intervention times are needed to demonstrate changes in health indicators such as reduced stunting. This overview paper summarises the programme and showcases examples from studies that demonstrate the impact pathway for nutrition interventions that encompass efficacy and effectiveness studies, value‐added processing, cost effectiveness of interventions, and bringing a proven intervention to scale.

**Key messages**

- Well‐planned research for nutrition interventions provides reliable new technical and social innovations, which should be tested considering contextual factors that might influence their effective delivery.
- Important to engage with policymakers and the private sector, especially when applying value‐added food fortification pathways.
- Scaling up nutrition‐sensitive agriculture programming requires multiple strategies and pathways to achieve sustainable results.
- Nutrition interventions that are also gender sensitive and have supportive environment to bring changes in capacity and behaviour of target population are needed.

## 1 BACKGROUND

Agriculture is central to human nutrition through its direct contributions to household food consumption, income generation, and women's empowerment (Haselow, Stormer, & Pries, 2016). There is a growing recognition that agricultural development is a strong entry point for efforts to improve nutrition with increased interest from donors and governments to leverage agriculture interventions to maximise nutritional impact. In 2017, it was estimated that 821 million people were undernourished, more than 1.5 billion were affected by micronutrient deficiencies, coupled with a worrying increase in the rate of overweight and obesity, with 38 million children under 5 years of age affected (Food and Agriculture Organization (FAO), International Fund for Agricultural Development, United Nations Children's Fund (UNICEF), World Food Programme, & World Health Organization, 2018).

Among the Sustainable Development Goals (SDGs) adopted by global leaders, SDG 2 aims to “end hunger, achieve food security and improved nutrition and promote sustainable agriculture” (United Nations, 2015). It identifies the linkages between agriculture interventions, empowering small‐holder farmers, promoting gender equality and healthy lifestyles, and tackling climate change. However, pathways from agriculture to improved nutrition are complex, encompassing economic, social, and gender considerations (McDermott, Johnson, Kadiyala, Kennedy, & Wyatt, 2015). Malnutrition is a multidimensional problem that requires multisectoral interventions. Nutrition‐ and gender‐sensitive agriculture provides timely and promising pathways to challenge the global problem of malnutrition.

## 2 CANADIAN INTERNATIONAL FOOD SECURITY RESEARCH FUND

People are considered food secure when they have access to sufficient, safe, nutritious food at all times in order to maintain a healthy and active life (FAO, 1996). The global food price crisis of 2007–2008 prompted the establishment of the Canadian International Food Security Research Fund (CIFSRF), a partnership between the International Development Research Centre (IDRC) and Global Affairs Canada (GAC) to contribute unique and innovative ideas to improve food and nutritional security, globally (IDRC, 2018). CIFSRF (2009–2018) was implemented in two phases supporting applied research to develop, test, and scale up promising food and nutrition security innovations. It invested CA$124.5 million in 39 projects that were selected through competitive calls and implemented in 25 countries by multistakeholder partnerships among Canadian universities and research institutions, the private and public sectors, and civil society groups from low‐ and middle‐income countries. From the beginning, the programme promoted nutrition‐based innovations alongside efforts to sustainably enhance food production by small‐holder farmers and increase their economic returns. To some degree, all projects addressed nutrition, but 10 projects had specifically identified nutrition related objectives.

During the first phase of CIFSRF (2009–2013), more than 144 food security‐based innovations were developed and field tested through a range of agriculture, food security, and nutrition projects. The second phase of CIFSRF (2013–2018) focused on scaling up 36 of the most promising innovations, reaching 78 million people. This includes people consuming better, healthier food, and farmers benefiting from improved income and productivity, reduced drudgery, and strengthened capacity. Details of contribution of CIFSRF to end global hunger and the strategies used have been synthesised at the conclusion of the programme (O'Neill & Manchur, 2018). CIFSRF built on IDRC's experience in supporting applied research for development and the programme strengths of GAC and leveraged the research capacity of Canadian and Southern institutions. The developers of the CIFSRF programme recognised that women and girls are less food secure than men and boys, despite being the main food producers in low‐income countries. It also identified women and girls as agents of change and targeted them as the main programme beneficiaries. By extension, all projects intentionally focused on gender by promoting gender equality and the empowerment of women and girls (IDRC, 2018). This strategy contributed to improved dietary diversity and nutrition for women and children, advanced economic development, impacted policy change, and is shaping the dialogue on food security within countries of implementation (Njuki, Parkins, & Kale, 2016).

The key elements that helped to achieve impact at scale during CIFSRF Phase 2 included generating evidence through rigorous research design to address specific challenges; selecting the most appropriate scaling up pathways to reach target populations for nutritional impact; responding to local context; identifying potential bottlenecks and opportunities for large scale adoption; selecting politically‐effective or influential partners; and investing in long‐term local capacity and leadership building (Shilomboleni & De Plaen, 2019).

To highlight results from specific projects that contributed to improved dietary diversity or filled a nutritional gap, a symposium at the 21st International Congress of Nutrition was organised in October 2017, with the theme of Research and Scaling Up Nutritionally Sensitive Agricultural Innovations. The symposium was attended by over 100 participants representing research and academic institutions, national and regional organizations, as well as bilateral, multilateral, and non‐governmental development partners. This special *Supplement* of Maternal and Child Nutrition includes selected presentations made at the symposium and other invited papers based on the importance of nutrition in their research. Several others are summarised in the synthesis report of the CIFSRF programme and include nutritious yellow potatoes from Columbia, aquaculture in Bolivia, indigenous vegetable‐fortified food products in West Africa, and cereal and pulse‐based complementary foods for children in Vietnam and Ethiopia (O'Neill & Manchur, 2018).

## 3 CIFSRF THEORY OF CHANGE FOR IMPROVED NUTRITION

**Figure 1:**
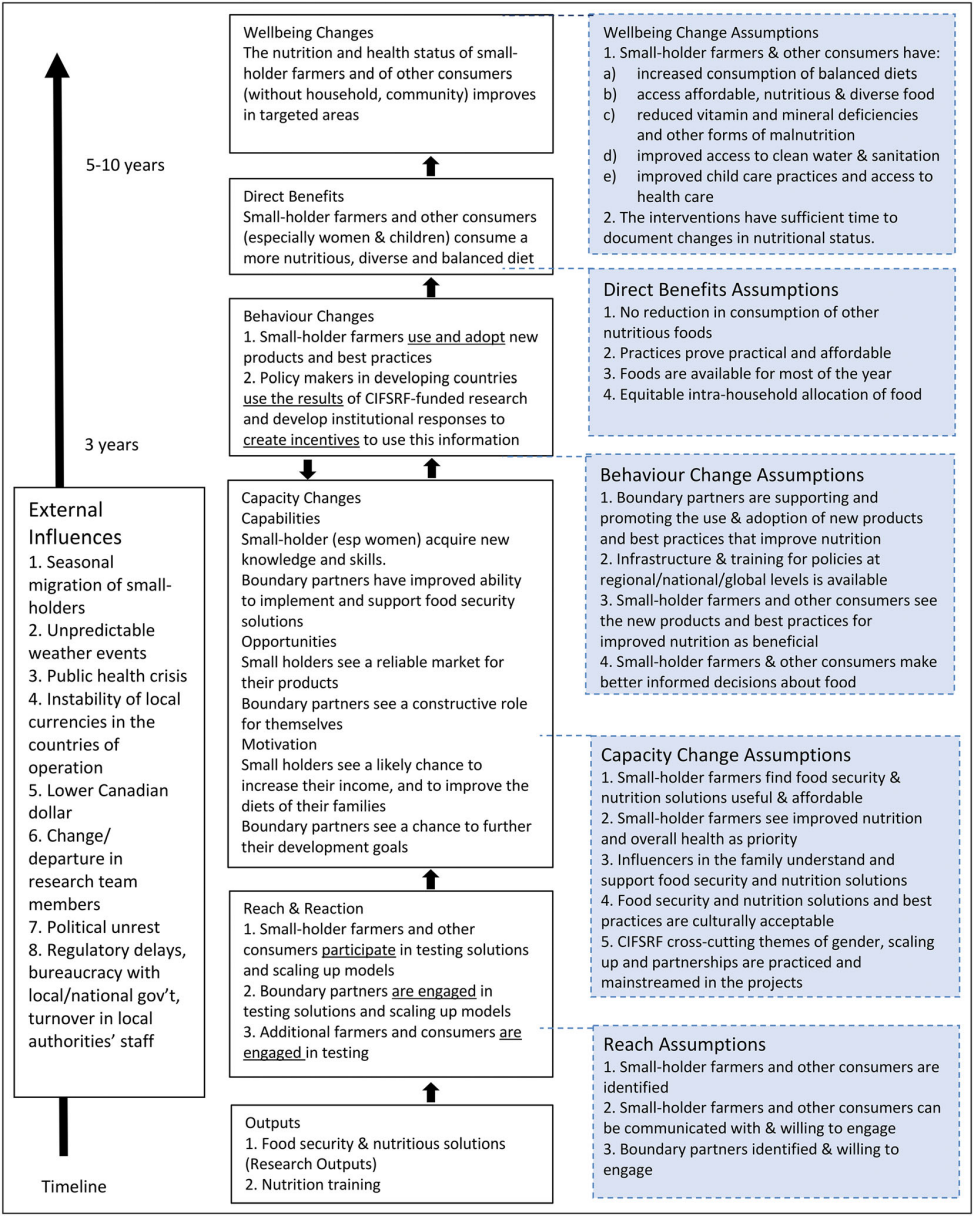
CIFSRF Theory of Change for improved nutrition

To understand the complex and varied means through which nutritional challenges can be addressed, a Theory of Change for improved nutrition was created. The Theory of Change for the CIFSRF programme was built using the method outlined by Mayne (2015) who suggests that enhanced capacity change in the target population is brought about through interventions that lead to behaviour change, which in turn improves well‐being (Mayne, 2015). Though the construction of the Theory of Change is both intuitive and sufficiently flexible to respond to the wide diversity of projects, it provided a structured framework and link to causality. The components of the Theory of Change are presented in Figure 1 and summarised in Box 1.

**Box 1: Components of the Theory of Change**

- Outputs—are practices and integrated models that improve dietary diversity, nutrition education and messages, value‐added processing methods, or specific fortification technologies.
- Reach and reaction—in the target population who are intended to receive the intervention's outputs and their initial reaction. The reach group here is food consumers, especially mothers and young children, small‐holder farmers and small to medium‐sized enterprises.
- Capacity changes—in one or more of knowledge, attitudes, skills, aspirations, and opportunities of those who have received or used the intervention's outputs.
- Behavioural changes—are alterations in actual practices that occur in the target population. An example is the changes in child feeding practices that occur as a result of the improved knowledge from training mothers. There, typically, is feedback between capacity and behavioural changes.
- Direct benefits—leading to improvements in the state of individual beneficiaries. This includes increased dietary diversity, consumption of nutritious diets by women and children, increased incomes, better use of health services, more productive farming, and women's empowerment.
- Well‐being changes—are longer term cumulative improvements such as better health, reduced poverty, and better food security. For example, improved diets should lead to better nutritional status.
- Assumptions—are events and conditions required for causal links to work and lead to desired effects. For example, for change to happen, it is assumed that the target group learns and implements solutions and that there is a positive enabling environment of the underlying determinants of nutrition.
- External influences—are factors not directly related to the intervention but that could contribute to the intended results or minimise their impact. As such, these factors could explain, in part, the success or failure of the intervention.

The complexity of the Theory of Change demonstrates how investing in agricultural production alone does not necessarily improve nutrition. Leveraging with market interventions, women's empowerment, and Behavior Change Communications to further improve availability of, access to, affordability of, and demand for nutritious foods is important (Ruel, Quisumbing, & Balagamwala, 2018). It also confirms how the complex interaction of food intake, water quality, care practices, disease burdens, sanitation, and health services drive a number of intermediate outcomes that affect nutrition, as do a range of deeper social, economic, and political processes (UNICEF, 2015).

## 4 SUMMARY OF SELECT NUTRITION‐SENSITIVE CIFSRF PROJECTS

**Figure 2:**
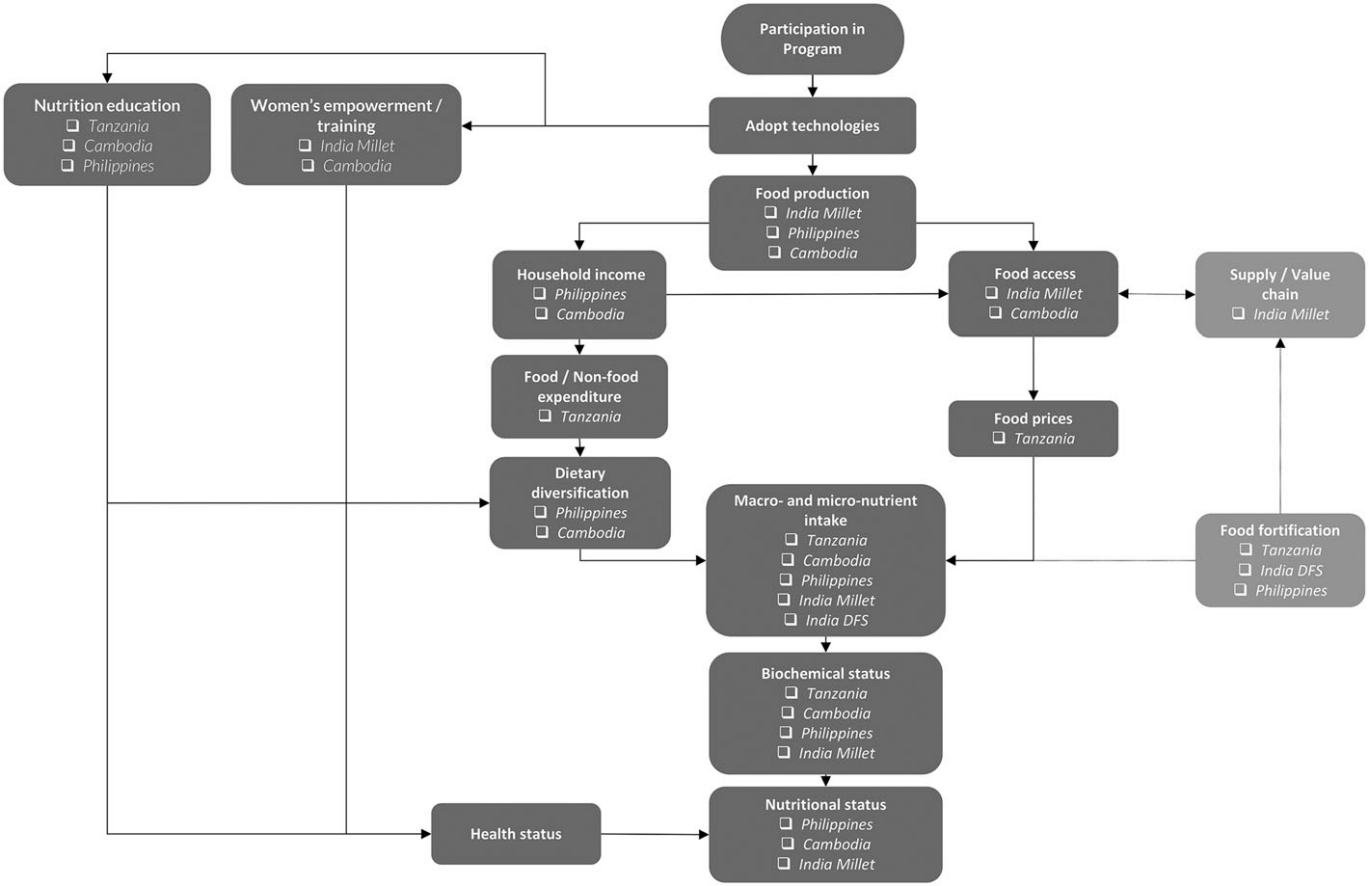
Agricultural interventions and nutrition outcomes: Simplified program impact pathway

The papers selected for this *Supplement* aim to improve health and well‐being of women and children through agriculture, nutrition, and food‐related research and interventions. The authors of the five papers demonstrate how a range of nutrition‐sensitive interventions, including home gardens and enhanced homestead food production, cropping improvements and diversification, and food fortification can be scaled up to contribute to the improvement of diets and nutritional outcomes in women and children. The papers provide evidence from efficacy (Michaux et al., 2019) and effectiveness (Angeles‐Agdeppa, Monville‐Oro, Gonsalves, & Capanzana, 2019) studies, to assessments of technologies to optimise value‐added processing (Durairaj, Gurumurthy, Nachimuthu, Muniappan, & Balasubramanian, 2019) and cost effectiveness of interventions (Walters, Ndau, Saleh, Mosha, & Horton, 2019), to a report of bringing a proven intervention to scale (Diosady, Mannar, & Krishnaswamy, 2019). These interventions aim to improve nutrition through a number of direct and indirect pathways, outlined in a simplified programme impact pathway adapted from Masset, Haddad, Cornelius, and Isaza‐Castro (2012; Figure 2). We have indicated how each of the five papers tackle food insecurity and malnutrition along this pathway within Figure 2 and have briefly summarised the projects below.

First, researchers in Cambodia conducted a rigorous randomised controlled trial to test the efficacy of an innovative nutrition‐sensitive agriculture programme of enhanced homestead food production, with the primary aim to reduce anaemia rates in women of reproductive age and their young children. While Helen Keller International, the local implementing partner, has implemented their homestead food production programme in Asia and Africa for over 20 years, the project in Cambodia was novel in that it included the addition of small‐scale polyculture (the raising of small and large indigenous fish together) to their standard programme of diversified home gardens instead of traditional small‐scale poultry production. This research team previously reported significant improvements in dietary intake in women and children among the study sample (Verbowski et al., 2018). However, as reported in the paper *Effect of enhanced homestead food production on anaemia among Cambodia women and children: A cluster randomized controlled trial*, although significant improvements were observed in anaemia rates among children in one of the intervention groups, no improvements were seen in women (Michaux et al., 2019). Although these findings are particularly promising for young children, they do not support the added benefit of polyculture plus diversified home gardens over diversified home gardens alone for women of reproductive age. The authors attribute the difference in impact between women and children to subsequent research that showed anaemia among women in Cambodia was likely not due to nutritional deficiencies (Wieringa et al., 2016) and thus a nutrition‐sensitive agricultural programme would have little impact on anemia in this population. Michaux et al. highlight the need to have a thorough understanding of the complex and ever‐changing causes of food insecurity and undernutrition in a population throughout the design stage and before implementation of a programme. This paper also demonstrates how critical it is to have end‐user buy‐in (i.e., small‐holder women farmers) for the scalability and sustainability of innovative programmes, as attrition was high and mostly due to work‐related migration, which was perhaps more attractive than the proposed solution.

Second, Angeles‐Agdeppa et al.'s (2019) *Integrated school based nutrition programme improved the knowledge of mother and schoolchildren* is a good example of an effectiveness evaluation. The authors report the implementation of an integrated school‐based nutrition programme that included gardening, nutrition education for parents, and supplementary feeding for children, and tested its impact on children's nutritional status. The basic premise of the paper demonstrates the relevance of situating an intervention in a sociocultural and political context as a prerequisite for implementing effective programmes. As explained in the paper, the integrated school‐based intervention model brought together the existing school garden and school nutrition programmes with supplementary feeding of malnourished children, which were being implemented disjointedly by separate government departments and added lessons from successful nutrition education for mothers. By testing and demonstrating the effectiveness of the integrated model in the practical setting, the authors found improved nutrition status of children. The authors also present a set of context‐appropriate strategies including nutritious menus for the supplementary feeding, use of iron fortified rice, appropriate nutrition messages for mothers, and best practices for school gardening that can be easily scaled up nationally because the policymakers in Philippines consider school‐based interventions as key national investments to improve children's health as a pathway to better food security and economy. With a number of countries identifying schools as an entry point for food security and nutrition interventions, a recent synthesis of experiences from eight countries point to the potential and complexity of linking local food production with the demand for nutritious foods created by school meal programmes (International Institute of Rural Reconstruction & IDRC, 2018).

Third, we included an example a novel technology to optimise value‐added processing with Durairaj et al.'s (2019) paper titled *Dehulled small millets: The promising nutricereals for improving the nutrition of children*. Small millets are a nutrient‐dense dietary staple but are consumed less often than other staples such as (micronutrient poor) paddy rice and wheat in India for various reasons, including suboptimal processing technology. In the context of high rates of malnutrition among children under 5 years in India (National Institution for Transforming India & Government of India, 2017), the aim of this project was to optimise dehulling as a means of incentivising the replacement of nutrient‐poor grains in the diet with small millets. These authors developed a novel double chamber centrifugal dehuller that resulted in more efficient Kernel recovery and dehulled small millets that retained the bran and endosperm layers and thus a majority of the nutrients. They then utilised the dehulled small millets in the development of a novel supplemental food that improved the height, weight, and haemoglobin concentrations of schoolchildren aged 4–6 years compared with control children not consuming a supplemental school meal. Muniappan et al.'s paper highlights the need for active industry engagement and buy‐in throughout the research process by leading readers through their series of industry consultations to identify current barriers to the small millet value chain in India and testing to ensure their technological solutions met the needs of small‐ and medium‐sized dehulling enterprises who would be the eventual users of the technology.

Fourth, Walters et al.'s (2019) paper titled *Cost‐effectiveness of sunflower oil fortification with vitamin A in Tanzania by scale* illustrates the costs and benefits of a vitamin A fortification programme in Tanzania. As described in the paper, the researchers leveraged the government‐mandated fortification of all large‐scale industrially produced edible oils with vitamin A, which had not yet met quality assurance standards for fortification levels, to implement an effectiveness study in two regions, whereby the researchers supported small‐ and medium‐scale oil producers process and distribute vitamin A fortified sunflower oil. The vitamin A status of women and children was then assessed to evaluate the nutritional impact of the programme. Although the results of the small‐ and medium‐scale fortification programme did not show clinically significant improvements in vitamin A status of women and children, the paper does demonstrate that the mandatory fortification programme of edible oils with vitamin A is a timely and economical solution for preventing morbidity and mortality among young children in Tanzania. Further, the paper shows that it is feasible and economical for small and medium producers to fortify sunflower oil with vitamin A, which could have a positive health impact on vulnerable women and children, if adequate coverage is ensured through increased government support and the necessary resources are provided. Analysing the costs and benefits of a programme, using a standard approach, is crucial to ensure sustainability and scalability of an innovation; it informs decision‐makers on how finite resources should be allocated for the largest impact (health or nutritional), particularly in low‐income countries where resources are scarce.

Finally, in the fifth paper, *Improving the lives of millions through new double fortification of salt technology*, Diosady et al. (2019) describe a complex journey from research consisting of efficacy and effectiveness studies, value chain development towards a cost‐effective solution and its scale up. They review their work spanning over 20 years towards a solution to address the persistent problem of iron deficiency in India, building on the success of salt iodization. The paper presents the rigorous research that identified the technology to encapsulate iron into a premix that can be safely added to iodised salt to produce double fortified salt (DFS) as a stable product. After a series of efficacy and effectiveness studies that proved that DFS can successfully address iron and iodine deficiencies among women and children, the authors demonstrate how the innovation from Canada was taken to Indian through transfer of technology to a local manufacturer. They then leveraged India's vast public distribution system, which targets the poor and reaches over 200 million people with essential commodities through its network of 500,000 fair price shops. The paper reports impressive results at scale by working in three Indian states where the government procures the locally manufactured DFS, which reached an estimated 60 million people in 2018. With high levels of iron deficiency in India affecting more than 50% of women and 70% of children, this paper also demonstrates the value of partnerships among researchers, policymakers, private sector, public sector, and the community to achieve a public health goal.

## 5 CONCLUSIONS

The lessons learned from the 39 CIFSRF projects in 25 countries demonstrate that projects with a specific focus on nutrition‐sensitive pathways can successfully change behaviours and diets and scale up production and consumption of nutritious foods. This was verified by an independent analysis of selected projects conducted in mid‐2018 (Wiggins, Keats, Löwe, & Shaxson, 2018). In the analysis, Wiggins et al. (2018) concluded that the nutrition‐focused CIFSRF programmes utilised different pathways to link agriculture interventions to nutritional outcomes. All projects worked towards greater inclusion of women at different stages of the supply and value chains to improve their decision‐making control within households. Most projects used a combination of three key strategies to improve nutrition and other health outcomes: dietary diversity and more nutritious crops; food fortification and value added‐processing; nutrition education and promotion. In this *Supplement*, we focus on the premise that nutrition‐ and gender‐sensitive agricultural and food‐based interventions have strong potential to improve the nutrition and health outcomes of the most vulnerable in low‐income settings, women of reproductive age and young children. Through the *Supplement*, we show that evidence of a successful intervention must stem from efficacy to effectiveness studies, must consider optimization of technologies and cost effectiveness and that truly scaled‐up solutions come from the culmination of these considerations along with strong government, non‐government organization, industry, and participant buy‐in. Although the results of these studies suggest that longer study durations and even intergenerational evaluations of interventions, may be needed to properly assess and confirm nutrition outcomes such as stunting, these papers add to the growing evidence suggesting that well‐designed nutrition‐sensitive agriculture and food‐based interventions can have meaningful impacts on the health and well‐being of women and children.

## CONFLICTS OF INTEREST

The authors declare that they have no conflicts of interest. The authors alone are responsible for the views expressed in this publication.

## CONTRIBUTIONS

ASW, KDM, and KCW did the primary drafting of this paper. All authors contributed to the conception and design of the paper, and reviewed and approved the overall paper.
